# Relationship between right and left ventricular diastolic dysfunction assessed by 2-dimensional speckle-tracking echocardiography in adults with repaired tetralogy of Fallot

**DOI:** 10.1007/s10554-020-02045-7

**Published:** 2020-10-02

**Authors:** Makoto Miyake, Rie Abe, Hayato Matsutani, Jiro Sakamoto, Hirokazu Kondo, Atsushi Iwakura, Hiraku Doi, Toshihiro Tamura

**Affiliations:** 1 Department of Cardiology, Tenri Hospital, Tenri, Japan; 2 Congenital Heart Disease Center, Tenri Hospital, Tenri, Japan; 3 Department of Clinical Laboratory, Tenri Hospital, Tenri, Japan; 4 Department of Cardiovascular Surgery, Tenri Hospital, Tenri, Japan; 5 Department of Cardiology, Congenital Heart Disease Center, Tenri Hospital, 200 Mishima-cho, 632-8552 Tenri, Japan

**Keywords:** Tetralogy of Fallot, Congenital heart disease, Diastolic dysfunction, Speckle-tracking echocardiography, Early diastolic strain rate

## Abstract

Several studies have reported a correlation between right ventricular (RV) and left ventricular (LV) systolic dysfunction in adults with repaired tetralogy of Fallot (TOF). However, data are lacking regarding the relationship between RV and LV diastolic dysfunction assessed by 2-dimensional speckle-tracking echocardiography. We studied 69 adults with repaired TOF (mean age 34 years, 61% male) who had been regularly followed up and had routinely undergone echocardiography. In addition to conventional echocardiography, global longitudinal strain (GLS) and early diastolic strain rate (SRe) of both ventricles were assessed using 2-dimensional speckle-tracking echocardiography. Results were compared with 30 age- and sex-matched controls. RV and LV GLS were decreased in TOF patients compared with controls (− 18.4 ± 3.3% vs. −23.5 ± 4.2%, p < 0.001 and − 16.0 ± 3.8% vs. −20.0 ± 3.0%, p < 0.001, respectively). RV and LV SRe were also decreased in TOF patients compared with controls (1.22 ± 0.34 sec^− 1^ vs. 1.47 ± 0.41 sec^− 1^, p = 0.003 and 1.29 ± 0.42 sec^− 1^ vs. 1.63 ± 0.42 sec^− 1^, p < 0.001, respectively). A correlation between RV and LV SRe was found in TOF patients (r = 0.43, p < 0.001) as well as between RV and LV GLS (r = 0.45, p < 0.001). Two-dimensional speckle-tracking echocardiography reveals subclinical RV and LV diastolic dysfunction in adults with repaired TOF. A correlation is observed between RV and LV diastolic dysfunction as well as between RV and LV systolic dysfunction.

## Introduction

Tetralogy of Fallot (TOF) is the most common cyanotic congenital heart disease [1]. Early surgical repair has dramatically improved long-term outcomes, and more than 90% of patients reach adulthood after repair of TOF [2, 3]. The right side of the heart has historically been paid attention in the management of patients late after repair of TOF [4–6].

However, recent studies have found that left ventricular (LV) systolic dysfunction is also associated with impaired clinical status in patients with repaired TOF [7] and is an independent predictor of adverse clinical events [8–10]. Decreased LV ejection fraction has been reported in 21% of patients with repaired TOF [11], and a study using speckle-tracking echocardiography revealed that subclinical LV systolic dysfunction was present even in TOF patients with normal LV ejection fraction [12]. Several studies have reported a correlation between right ventricular (RV) and LV systolic dysfunction in patients with repaired TOF, indicating ventricular interaction in which RV systolic dysfunction adversely affects LV systolic function [7, 12–14].

LV diastolic dysfunction in patients with repaired TOF have also been investigated [15–19]; however, data are lacking regarding the relationship between RV and LV diastolic dysfunction assessed by 2-dimensional speckle-tracking echocardiography. We hypothesized that there would also be a correlation between subtle RV and LV diastolic dysfunction in adults with repaired TOF. This study investigated the relationship between RV and LV diastolic dysfunction in addition to the relationship between RV and LV systolic dysfunction in asymptomatic adults with repaired TOF using 2-dimensional speckle-tracking echocardiography.

## Methods

### Study population

This was a single-center, retrospective, cross-sectional study. The study included asymptomatic adults (age ≥ 15 years) with repaired TOF, who had received regular follow-up at Congenital Heart Disease Center, Tenri Hospital (Tenri, Japan) and had routinely undergone echocardiography between January 2012 and August 2018. We used an echocardiographic database to screen for eligible patients with repaired TOF. Only echocardiography performed with Vivid 7 or Vivid E9 machine (GE Healthcare, Horten, Norway) was included to avoid variability in strain measurements between different vendors. For those who had undergone several echocardiographic examinations with the GE machines during this period, the most recent one was used for analysis. Exclusion criteria were as follows: echocardiography without the entire RV free wall in the apical 4-chamber view, poor image quality for speckle-tracking analysis, and patients who were not in sinus rhythm. Echocardiographic data were compared with those of 30 age- and sex-matched normal controls.

This study was conducted in accordance with the Declaration of Helsinki. The institutional ethics committee at Tenri Hospital approved the protocol (Approval number 1072). Written informed consent was waived because of the retrospective nature of the study.

### Conventional echocardiography

All echocardiography was performed by experienced sonographers according to standardized protocols using Vivid 7 or Vivid E9 machines (GE Healthcare). Images were acquired from standard parasternal and apical views and digitally stored. Measurements included RV end-diastolic and end-systolic area, RV fractional area change, right atrial end-systolic area, LV end-diastolic and end-systolic dimeter, LV ejection fraction (using biplane Simpson’s method), LV wall thickness, left atrial volume, and tricuspid annular plane systolic excursion (TAPSE). Severity of pulmonary regurgitation (PR) was graded by jet width, length, and duration using Doppler echocardiography according to the American Society of Echocardiography (ASE) and the European Association of Cardiovascular Imaging recommendations [20]. Severity of pulmonary stenosis (PS) was graded by peak velocity of the jet across the narrowing lesion. Peak velocity of more than 4 m/s and 3–4 m/s were considered severe PS and moderate PS, respectively, according to current American College of Cardiology/American Heart Association guidelines [21]. Doppler measurements also included transmitral flow velocity ratio (E/A), diastolic tricuspid (tricuspid E′) and mitral lateral annular velocity (mitral E′).

### Speckle-tracking echocardiography

**Fig. 1:**
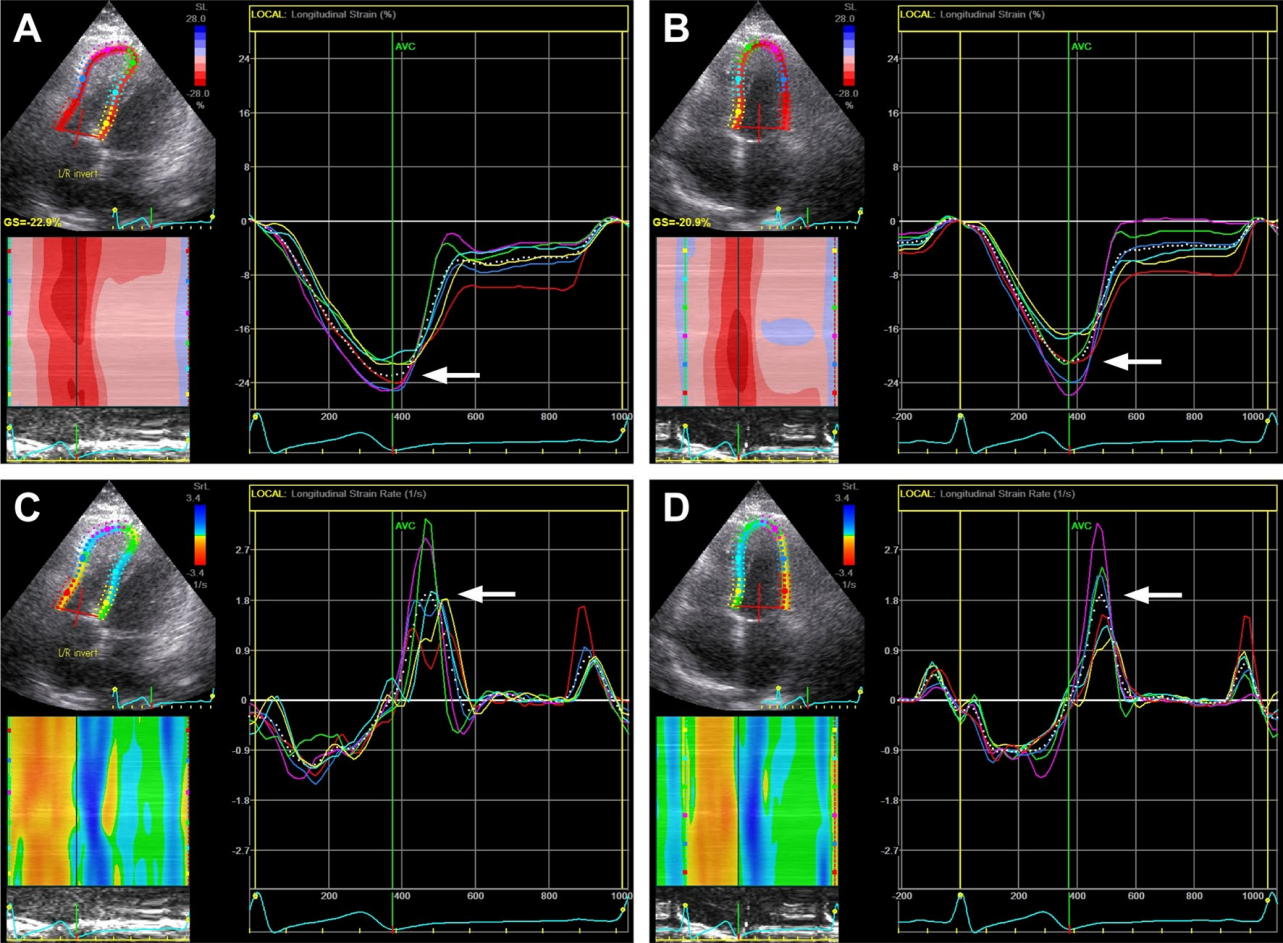
Two-dimensional speckle-tracking echocardiography in the apical 4-chamber view. White dotted lines indicate GLS or global longitudinal strain rate (average of all segments). White arrow indicates RV GLS (**a**), LV GLS (**b**), RV SRe (**c**), LV SRe (**d**). *GLS* global longitudinal strain, *LV* left ventricular, *RV* right ventricular, *SRe* early diastolic strain rate

RV and LV strain and strain rate were analyzed offline using 2-dimensional speckle-tracking analysis software (EchoPAC version 113.1.0; GE Healthcare). The LV algorithm was used for both ventricles. The endocardial border was traced in the apical 4-chamber view and the width of the region of interest was adjusted. Myocardial tracking was confirmed visually and verified with the software. The endocardial border was retraced if myocardial tracking was inadequate. Peak systolic longitudinal strain was defined as the peak negative value on the curve. As in LV global longitudinal strain (GLS) analysis, RV GLS was defined as the average of peak systolic longitudinal strain of all segments of the right ventricle including the ventricular septum [22]. RV and LV early diastolic strain rate (SRe) were defined as the average of peak early diastolic longitudinal strain rate of all segments of the right and left ventricle, respectively. All images were recorded at a frame rate of more than 50 frames/sec. Representative GLS and SRe curves for both ventricles in a patient with repaired TOF are shown in Fig. 1.

### Statistical analysis

Categorical variables were presented as numbers and percentages and compared using the chi-squared test. Continuous variables were expressed as mean and standard deviation or median and interquartile range and compared using the Student's *t*-test or Wilcoxon rank sum test based on their distributions. Relationships between two continuous variables were assessed using Pearson’s correlation analysis. Variables which were not normally distributed were log transformed. Intra- and interobserver reproducibility for conventional and speckle-tracking echocardiographic parameters of both ventricles were assessed by calculating interclass correlation coefficient in 20 randomly selected patients with repaired TOF by the same observer and two different observers, respectively. All statistical analyses were conducted with SPSS software version 22.0 (IBM Corp., Armonk, New York). All reported p values were two-tailed, and p < 0.05 were considered statistically significant.

## Results

### Study population and feasibility of echocardiographic measurements

A total of 113 adult patients with repaired TOF were identified to be potentially eligible for enrollment using the echocardiographic database. Among them, RV speckle-tracking analysis was not feasible in 44 patients because the entire RV wall was not recorded in the apical 4-chamber view or image quality was too poor to perform adequate myocardial tracking. LV speckle-tracking analysis was not feasible in 21 patients because of poor image quality. The feasibility of RV speckle-tracking analysis was 61%, which was lower than that of measuring TAPSE (86%, p = 0.004) and tricuspid E′ (88%, p = 0.007). The feasibility of LV speckle-tracking analysis was 81%, which was also lower than that of measuring LV EF (88%, p < 0.001) and mitral E′ (94%, p = 0.010). This study finally included 69 TOF patients in which biventricular speckle-tracking analysis was feasible. Sixty-three patients had undergone intracardiac repair at our hospital; the remaining 6 patients had undergone this surgery at other hospitals.

### Patients characteristics

Demographic and clinical characteristics are presented in Table 1. Median age at initial intracardiac repair was 39 months. Five patients (7%) underwent initial repair at the age of ≥ 15 years. Median time from initial intracardiac repair was 30 years. One patient underwent pulmonary valve replacement at initial repair and 11 patients underwent pulmonary valve replacement after repair. QRS duration was more prolonged in patients with repaired TOF than controls.

**Table 1:** Clinical characteristics

	Patients (n = 69)	Controls (n = 30)	p value
Age, years	34 ± 13	36 ± 11	0.38
Male	42 (61)	17 (57)	0.70
BSA, m^2^	1.61 ± 0.18	1.68 ± 0.20	0.09
Prior palliative procedures	16 (23)	–	–
Age at repair, months	39 (20–52)	–	–
Type of repair			
Valve-sparing	34 (49)	–	–
Transannular patch	30 (43)	–	–
Pulmonary valve replacement	1 (1)	–	–
Unknown	4 (6)	–	–
Time from repair, years	30 (20–37)	–	–
Pulmonary valve replacement	12 (17)	–	–
Number of prior open heart surgery	1 (1–2)	–	–
Heart rate, bpm	63 ± 10	63 ± 10	0.90
Systolic blood pressure, mmHg	121 ± 18	124 ± 20	0.51
Diastolic blood pressure, mmHg	69 ± 10	72 ± 15	0.32
QRS duration, msec	136 ± 29	94 ± 10	< 0.001

Values are presented as mean ± SD, number (%), or median (interquartile range)

*BSA* body surface area

### Conventional echocardiography

Conventional echocardiographic findings are presented in Table 2. RV end-diastolic area and right atrial end-systolic area were larger, and LV end-diastolic diameter was smaller in patients with repaired TOF than controls. Five patients (7%) had decreased RV fractional area change (< 35%). One patient (1.4%) had moderately decreased LV ejection fraction (< 45%), 10 patients (14%) had mildly decreased LV ejection fraction (< 55%).

**Table 2:** Conventional echocardiographic findings

	Patients (n = 69)	Controls (n = 30)	p value
RV end-diastolic area, cm^2^	25.1 ± 6.8	16.9 ± 3.7	< 0.001
RV end-systolic area, cm^2^	14.5 ± 4.3	9.2 ± 2.1	< 0.001
RV FAC, %	42.5 ± 5.9	45.5 ± 4.6	0.013
RA end-systolic area, cm^2^	16.9 ± 5.7	12.5 ± 2.0	< 0.001
LV EDD, mm	43.3 ± 4.7	46.1 ± 3.3	0.004
LV ESD, mm	28.9 ± 4.6	29.6 ± 3.3	0.48
LV EF, %	61.9 ± 6.6	65.9 ± 4.9	0.005
IVS thickness, mm	9.2 ± 1.7	8.8 ± 0.9	0.17
PW thickness, mm	8.8 ± 1.4	8.8 ± 1.0	0.92
LA volume, ml	49.7 ± 14.5	49.3 ± 8.7	0.89
TAPSE, mm	15.2 ± 3.5	23.3 ± 2.9	< 0.001
Moderate to severe PR	26 (38)	0	< 0.001
Moderate to severe PS	12 (17)	0	< 0.001
Moderate to severe TR	4 (6)	0	0.43
TMF E/A	2.08 ± 0.82	1.83 ± 0.79	0.18
Mitral E′, cm/s	13.1 ± 3.9	14.3 ± 3.2	0.15
Mitral E/E′	7.7 ± 3.3	5.9 ± 1.5	0.006
Tricuspid E′, cm/s	9.7 ± 3.9	12.7 ± 2.5	< 0.001

Values are presented as mean ± SD or number (%).

*EDD* end-diastolic diameter, *EF* ejection fraction, *ESD* end-systolic diameter, *FAC* fractional area change, *IVS* interventricular septum, *LA* left atrial, *LV* left ventricular, *PR* pulmonary regurgitation, *PS* pulmonary stenosis, *PW* posterior wall, *RA* right atrial, *RV* right ventricular, *TAPSE* tricuspid annular plane systolic excursion, *TMF* transmitral flow, *TR* tricuspid regurgitation

### Global longitudinal strain

**Fig. 2:**
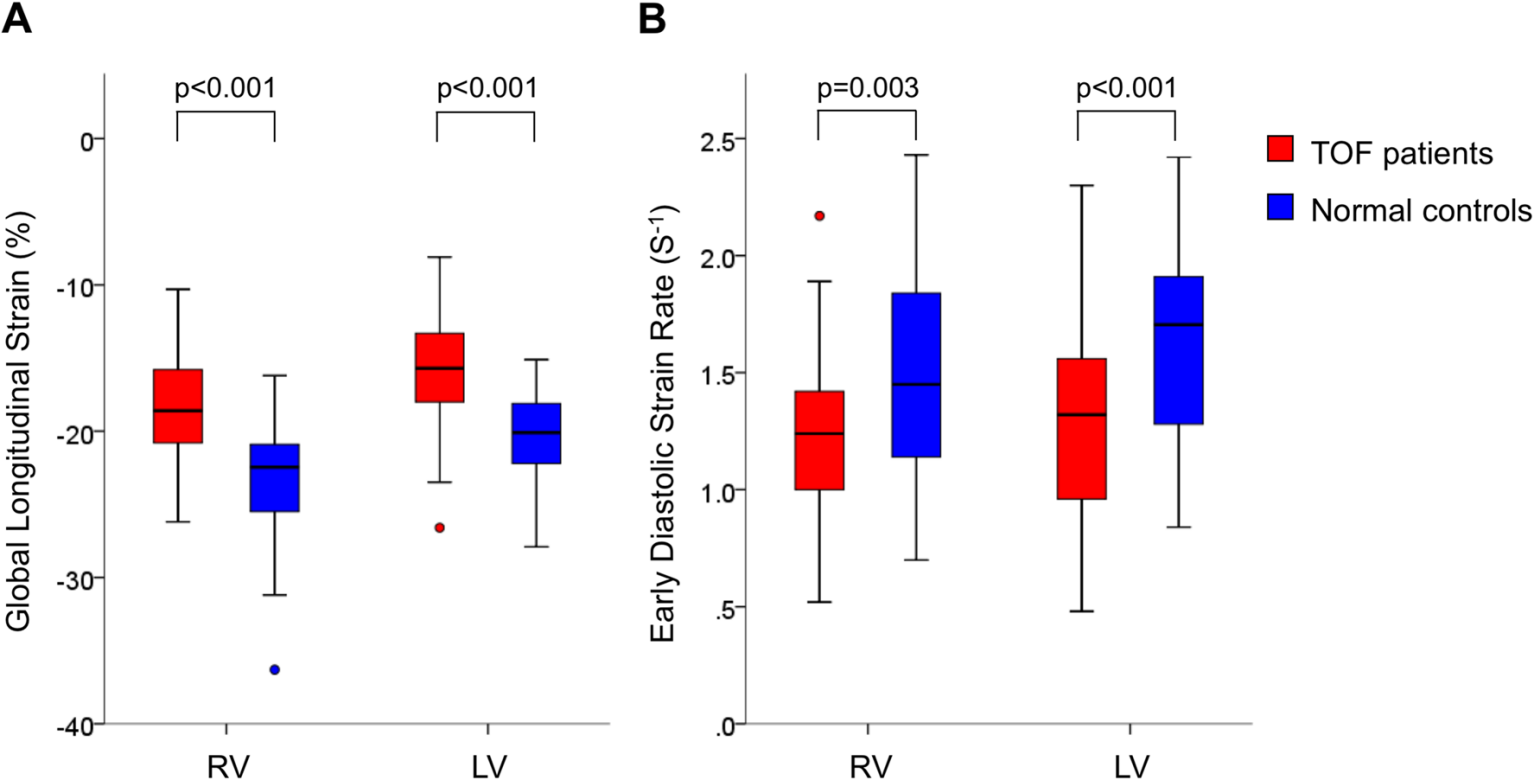
Comparison of global longitudinal strain (**a**) and early diastolic strain rate (**b**) between patients with repaired TOF and controls. *LV* left ventricle, *RV* right ventricle, *TOF* tetralogy of Fallot

RV and LV GLS were significantly decreased in patients with repaired TOF compared with controls (− 18.4 ± 3.3% vs. −23.5 ± 4.2%, p < 0.001 and − 16.0 ± 3.8% vs. −20.0 ± 3.0%, p < 0.001, respectively; Fig. 2a).

RV and LV GLS did not significantly correlate with age at time of imaging (r = 0.14, p = 0.25 and r = 0.16, p = 0.19, respectively) or log-transformed age at repair (r = 0.11, p = 0.38 and r = 0.02, p = 0.88, respectively). RV and LV GLS were not significantly different between TOF patients with and without moderate to severe PR (− 18.8 ± 3.0% vs. −18.1 ± 3.5%, p = 0.39 and − 15.5 ± 3.6% vs. −16.3 ± 3.9%, p = 0.43, respectively) and between those with and without moderate to severe PS (− 18.1 ± 3.7% vs. −18.4 ± 3.3%, p = 0.76 and − 16.6 ± 4.6% vs. −15.9 ± 3.6%, p = 0.57, respectively). RV and LV GLS correlated with QRS duration in patients with repaired TOF (r = 0.24, p = 0.045 and r = 0.41, p < 0.001, respectively). RV GLS correlated with TAPSE (r = − 0.43, p < 0.001) and LV GLS correlated with LV EF (r = − 0.41, p = 0.002) in patients with repaired TOF.

**Fig. 3:**
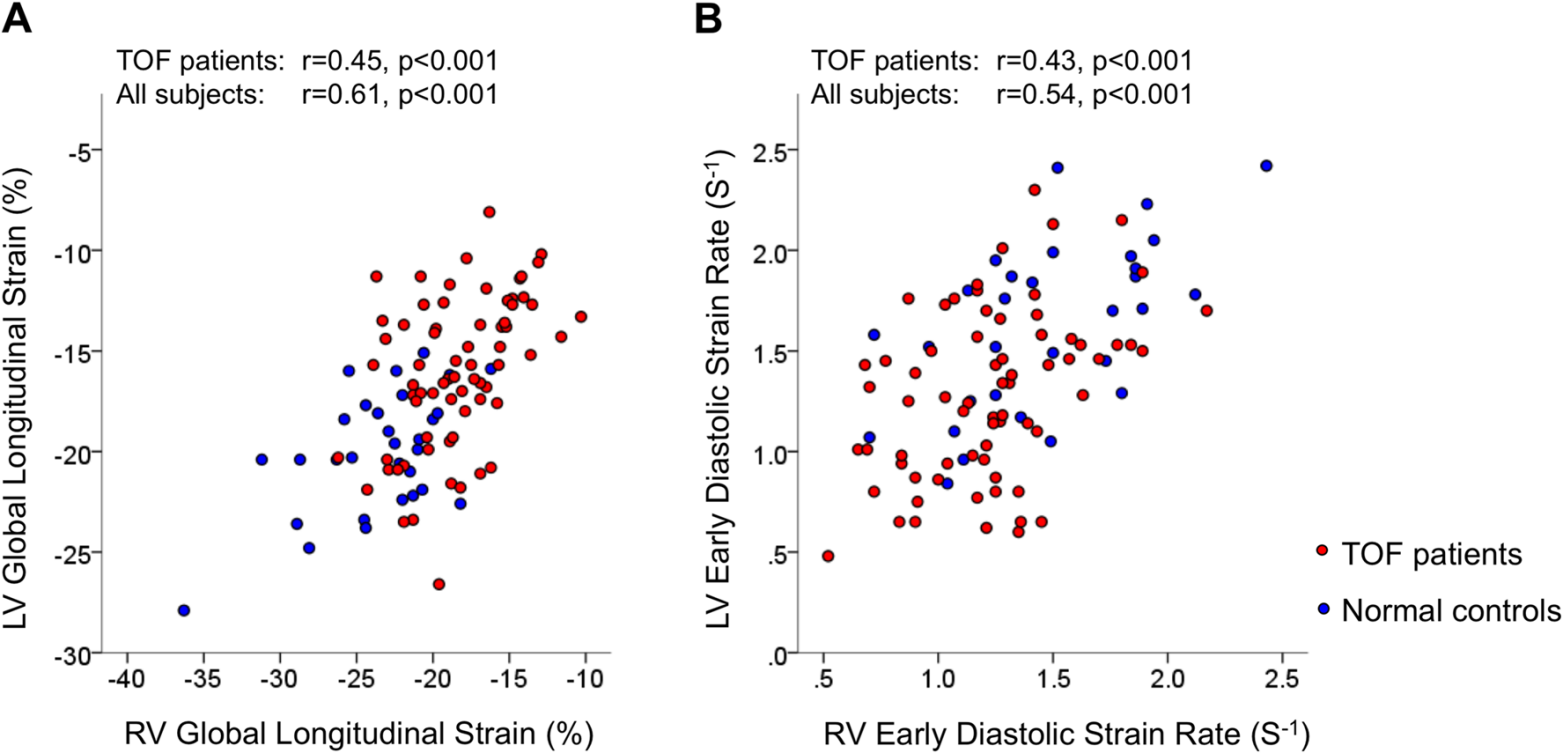
Correlations between RV and LV global longitudinal strain (**a**) and between RV and LV early diastolic strain rate (**b**) . *LV* left ventricular, *RV* right ventricular, *TOF* tetralogy of Fallot

A correlation between RV GLS and LV GLS was found in patients with repaired TOF (r = 0.45, p < 0.001; Fig. 3a) as with controls (r = 0.55, p = 0.002).

### Early diastolic strain rate

RV and LV SRe were significantly decreased in patients with repaired TOF compared with controls (1.22 ± 0.34 sec^− 1^ vs. 1.47 ± 0.41 sec^− 1^, p = 0.003 and 1.29 ± 0.42 sec^− 1^ vs. 1.63 ± 0.42 sec^− 1^, p < 0.001, respectively; Fig. 2b).

RV and LV SRe correlated with age at time of imaging (r = − 0.39, p < 0.001 and r = − 0.46, p < 0.001, respectively) and log-transformed age at repair (r = − 0.31, p = 0.012 and r = − 0.30, p = 0.012, respectively). RV and LV SRe were not significantly different between TOF patients with and without moderate to severe PR (1.29 ± 0.31% vs. 1.19 ± 0.35%, p = 0.26 and 1.32 ± 0.47% vs. 1.27 ± 0.40%, p = 0.64, respectively) and between those with and without moderate to severe PS (1.18 ± 0.31% vs. 1.24 ± 0.34%, p = 0.59 and 1.33 ± 0.40% vs. 1.28 ± 0.43%, p = 0.71, respectively). RV and LV SRe correlated with QRS duration in patients with repaired TOF (r = − 0.31, p = 0.010 and r = − 0.25, p = 0.038, respectively). RV SRe correlated with tricuspid E′ (r = 0.51, p < 0.001) and LV SRe correlated with mitral E′ (r = 0.46, p < 0.001) in patients with repaired TOF.

A correlation between RV SRe and LV SRe was found in patients with repaired TOF (r = 0.43, p < 0.001; Fig. 3b) as with controls (r = 0.59, p < 0.001).

### Reproducibility

The intra- and interobserver interclass correlation coefficient were 0.92 (95% confidence interval [CI] 0.82–0.97) and 0.86 (95% CI 0.55–0.95) for TAPSE, respectively, and 0.97 (95% CI 0.92–0.99) and 0.91 (95% CI 0.79–0.96) for LV EF, respectively. Reproducibility of tricuspid and mitral E′ could not be investigated because video clips of tissue Doppler imaging are not routinely stored at our hospital. The interclass correlation coefficient for GLS and SRe of both ventricles are presented in Table 3. All parameters showed good intra- and interobserver reproducibility with interclass correlation coefficients of > 0.8.

**Table 3:** Reproducibility of speckle-tracking analysis

Variable	Intraobserver ICC (95% CI)	Interobserver ICC (95% CI)	
RV GLS	0.92 (0.80–0.97)	0.90 (0.76–0.96)	
LV GLS	0.98 (0.95–0.99)	0.92 (0.81–0.97)	
RV SRe	0.94 (0.83–0.97)	0.87 (0.69–0.95)	
LV SRe	0.95 (0.89–0.98)	0.94 (0.85–0.98)	

*CI* confidence interval, *GLS* global longitudinal strain, *ICC* interclass correlation coefficient, *LV* left ventricular, *RV* right ventricular, *SRe* early diastolic strain rate

## Discussion

This study investigated subclinical RV and LV systolic and diastolic dysfunction in adults with repaired TOF using 2-dimensional speckle-tracking echocardiography. The main results were as follows: (1) 2-dimensional speckle-tracking echocardiography was able to reveal a decrease in RV and LV SRe in asymptomatic adults with repaired TOF compared with controls; (2) a correlation was observed between RV and LV SRe as well as between RV and LV GLS.

Recently, SRe has been proposed as a more accurate marker of myocardial relaxation than conventional tissue Doppler echocardiography [23, 24]. Strain rates are not angle dependent, not affected by myocardial tethering, and are less affected by loading condition [23]. Its usefulness for assessing LV diastolic function has been reported in general population [25], patients with valvular heart disease [26], and patients with coronary artery disease [27]. In our study, despite a decrease in LV SRe in patients with repaired TOF, left atrial volume, mitral E/A, and mitral E′ were not significantly different between patients with repaired TOF and controls, and mitral E/E′ in patients with repaired TOF was slightly increased but remained within normal range. A previous study also showed that Doppler measurements were not associated with elevated LV end-diastolic pressure (> 12 mmHg) in patients with repaired TOF [19]. These results suggest that LV SRe decreases at an earlier stage of LV diastolic dysfunction, in which conventional echocardiographic parameters do not yet indicate the presence of LV diastolic dysfunction. Thus, LV SRe appears to be a more sensitive marker to detect subtle LV diastolic dysfunction in patients with repaired TOF.

There are no established parameters regarding assessment of RV diastolic dysfunction in patients with repaired TOF. In our study, tricuspid E′ was decreased in patients with TOF. However, previous studies have reported that conventional parameters using tricuspid flow velocities and tricuspid annular velocities were not associated with RV end-diastolic pressure in children with repaired TOF [28, 29]. Furthermore, the ASE guidelines for imaging in repaired TOF state that Doppler parameters of tricuspid inflow are not reliable for assessment of RV diastolic function [30]. Meanwhile, speckle-tracking analysis has recently become feasible in not only the left ventricle but also the right ventricle [22]. Our study suggests that RV SRe has a potential to become a novel marker of RV diastolic function in patients with repaired TOF.

The mechanism of LV diastolic dysfunction in adults with repaired TOF is not fully understood; in particular, data on its relationship with RV diastolic dysfunction assessed by 2-dimensional speckle-tracking echocardiography are lacking. A previous study investigated RV and LV diastolic dysfunction in children (mean age 12 years) with repaired TOF using speckle-tracking echocardiography [17]. This previous study demonstrated that RV SRe in children with repaired TOF was decreased compared with controls, whereas LV SRe was not significantly different. Based on this previous study and our results, we speculate that RV diastolic dysfunction has a long-term adverse effect on LV diastolic function after repair of TOF.

Our study suggests that ventricular interaction plays a role in diastolic dysfunction, as has previously been reported for systolic dysfunction. Recent studies with cardiac magnetic resonance in patients with repaired TOF found a correlation between RV and LV extracellular volume fraction [31] and between RV and LV native T1 value [32], suggesting that the degree of LV fibrosis correlates with that of RV fibrosis. These results can explain impairment of both RV and LV contractility and relaxation, and correlation of systolic and diastolic dysfunction between the right and left ventricles, as shown in our study.

The mechanism of ventricular interaction remains unclear. Mechanical coupling may provide an explanation. The right and left ventricles share a continuous myocardial band [33]. Impaired RV myocardial performance may adversely affect LV myocardial performance through this shared myocardial band. Neurohormonal coupling may also provide an explanation for ventricular interaction. Neurohumoral activation or inhibition has been reported to alter myocardial relaxation and stiffness [34], and neurohormonal activation has been reported to exist in adults with congenital heart disease [35]. Genetic factors have been reported to be associated with RV dysfunction in patients with TOF [36]. Given that TOF is a congenital disease, genetic factors may also influence LV remodeling as with RV remodeling.

## Limitations

This study has several limitations. First, this is a retrospective study, therefore there is a potential for selection bias. Not all echocardiographic images of eligible patients from the echocardiographic database could be analyzed due to the inability to track the RV myocardium. Second, this study has demonstrated low feasibility of speckle-tracking echocardiography for the assessment of RV function in patients with repaired TOF. However, this limitation may be resolved if more attention is paid to acquisition of good-quality images of the entire right ventricle. Third, all patients in the study were clinically stable and did not undergo cardiac catheterization around the time of echocardiography; therefore, we could not directly compare the accuracy of SRe and E′ using catheter-derived references such as relaxation time constant (Tau), peak negative dP/dt, or end-diastolic pressure. Fourth, RV assessment by 2-dimensional speckle-tracking echocardiography did not include RV outflow tract function. Previous studies have suggested that RV outflow tract systolic dysfunction is related to impairment of RV performance in patients with repaired TOF [37, 38]. RV outflow tract may also contribute to RV diastolic function, although RV outflow tract is a relatively small portion compared to the inlet and trabecular portion. Fifth, the subgroup analysis by PR and PS showed no significant difference in GLS and SRe. This may be due to a lack of power because of small sample size. Finally, because this is a cross-sectional study, we could not demonstrate association between subclinical diastolic dysfunction detected by 2-dimensional speckle-tracking echocardiography and long-term clinical outcomes. Further research is needed to assess whether decreased RV and LV SRe are associated with poor clinical outcomes.

## Conclusions

Two-dimensional speckle-tracking echocardiography reveals subclinical RV and LV diastolic dysfunction in adults with repaired TOF. A correlation is observed between RV and LV diastolic dysfunction as well as between RV and LV systolic dysfunction in adults with repaired TOF.
