# Correction to: Relationship between right and left ventricular diastolic dysfunction assessed by 2-dimensional speckle-tracking echocardiography in adults with repaired tetralogy of Fallot

**DOI:** 10.1007/s10554-021-02473-z

**Published:** 2021-12-23

**Authors:** Makoto Miyake, Rie Abe, Hayato Matsutani, Jiro Sakamoto, Hirokazu Kondo, Atsushi Iwakura, Hiraku Doi, Toshihiro Tamura

**Affiliations:** 1 Department of Cardiology, Tenri Hospital, Tenri, Japan; 2 Congenital Heart Disease Center, Tenri Hospital, Tenri, Japan; 3 Department of Clinical Laboratory, Tenri Hospital, Tenri, Japan; 4 Department of Cardiovascular Surgery, Tenri Hospital, Tenri, Japan; 5 Department of Cardiology, Congenital Heart Disease Center, Tenri Hospital, 200 Mishima‑cho, Tenri, 632‑8552 Japan

## Correction to: The International Journal of Cardiovascular Imaging (2021) 37:569–576 10.1007/s10554-020-02045-7

The article “Relationship between right and left ventricular diastolic dysfunction assessed by 2-dimensional speckle-tracking echocardiography in adults with repaired tetralogy of Fallot written by Makoto Miyake · Rie Abe · Hayato Matsutani · Jiro Sakamoto · Hirokazu Kondo · Atsushi Iwakura · Hiraku Doi · Toshihiro Tamura”, was originally published Online First without Open Access. After publication in volume 37, issue 2, page 569–576 the author decided to opt for Open Choice and to make the article an Open Access publication. Therefore, the copyright of the article has been changed to © The Author(s) 2021 and the article is forthwith distributed under a Creative Commons Attribution 4.0 International License, which permits use, sharing, adaptation, distribution and reproduction in any medium or format, as long as you give appropriate credit to the original author(s) and the source, provide a link to the Creative Commons licence, and indicate if changes were made. The images or other third party material in this article are included in the article’s Creative Commons licence, unless indicated otherwise in a credit line to the material. If material is not included in the article’s Creative Commons licence and your intended use is not permitted by statutory regulation or exceeds the permitted use, you will need to obtain permission directly from the copyright holder. To view a copy of this licence, visit https://creativecommons.org/licenses/by/4.0.

The original article has been corrected.

